# Blastic plasmacytoid dendritic cell neoplasm frequently shows occult central nervous system involvement at diagnosis and benefits from intrathecal therapy

**DOI:** 10.18632/oncotarget.7101

**Published:** 2016-01-31

**Authors:** Lourdes Martín-Martín, Julia Almeida, Helena Pomares, Eva González-Barca, Pilar Bravo, Teresa Giménez, Cecilia Heras, José-Antonio Queizán, Elena Pérez-Ceballos, Violeta Martínez, Natalia Alonso, Carlota Calvo, Rodolfo Álvarez, María Dolores Caballero, Alberto Orfao

**Affiliations:** ^1^ Cancer Research Centre (IBMCC, USAL-CSIC), Institute for Biomedical Research of Salamanca (IBSAL) and Department of Medicine and Cytometry Service (NUCLEUS Research Support Platform), University of Salamanca (USAL), Salamanca, Spain; ^2^ Hematology Department, Institut Catalá d'Oncologia, Hospital Duran i Reynals, University of Barcelona, Institut d'Investigació Biomèdica de Bellvitge, Barcelona, Spain; ^3^ Hematology Department, University Hospital of Fuenlabrada, Madrid, Spain; ^4^ Hematology Department, University Hospital Joan XXIII, Tarragona, Spain; ^5^ Hematology Department, Infanta Leonor Hospital, Madrid, Spain; ^6^ Hematology Department, Hospital of Segovia, Segovia, Spain; ^7^ Hematology Department, University Hospital Morales Meseguer, Murcia, Spain; ^8^ Hematology Department, Hospital of León, León, Spain; ^9^ Hematology Department, University Hospital of Santiago, Santiago de Compostela, Spain; ^10^ Hematology Department, University Hospital Miguel Servet, Zaragoza, Spain; ^11^ Hematology Department, General Yagüe Hospital, Burgos, Spain; ^12^ Hematology Department and IBSAL, University Hospital of Salamanca, Salamanca, Spain

**Keywords:** blastic plasmacytoid dendritic cell neoplasm, central nervous system, intrathecal prophylaxis, ALL therapy, flow cytometry

## Abstract

Blastic plasmacytoid dendritic cell neoplasm (BPDCN) is a rare aggressive myeloid neoplasm which shows a high rate of central nervous system (CNS) recurrence and overall survival (OS) of <1 year. Despite this, screening for CNS involvement is not routinely performed at diagnosis and intrathecal (IT) prophylaxis is not regularly administered in BPDCN. Here, we prospectively evaluated 13 consecutive BPDCN patients for the presence of CNS involvement by flow cytometry. Despite none of the patients presented with neurological symptoms, occult CNS involvement was detected in 6/10 cases evaluated at diagnosis and 3/3 studied at relapse/progression. BPDCN patients evaluated at diagnosis received IT treatment -either CNS prophylaxis (*n* = 4) or active therapy (*n* = 6)- and all but one remain alive (median follow-up of 20 months). In contrast, all three patients assessed at relapse/progression died. The potential benefit of IT treatment administered early at diagnosis on OS and CNS recurrence-free survival of BPDCN was further confirmed in a retrospective cohort of another 23 BPDCN patients. Our results show that BPDCN patients studied at diagnosis frequently display occult CNS involvement; moreover, they also indicate that treatment of occult CNS disease might lead to a dramatically improved outcome of BPDCN.

## INTRODUCTION

Blastic plasmacytoid dendritic cell neoplasm (BPDCN) is a rare disease -i.e. < 1% acute myeloblastic leukemia cases (AML)- with a historically dismal prognosis. Despite most BPDCN patients treated with different intensive chemotherapy regimens (e.g. CHOP, AML-type and acute lymphoblastic leukemia (ALL)-type regimens) achieve complete remission (CR), the great majority relapse early after CR, with further resistance to therapy and a fatal outcome. Of note, a significant percentage (≈30%) [1-3] of BPDCN relapses occur in the central nervous system (CNS), either as isolated CNS relapses or in the context of a systemic relapse. This is in line with the relatively high rate (≈10% of cases) [1-6] of overt CNS involvement (i.e. confirmed by cytology) observed at diagnosis in patients with leukemic variant of BPDCN presenting with neurological symptoms.

Although BPDCN prognosis is extremely poor, patients treated with ALL-type regimens that include CNS prophylaxis [1-3] and/or allogeneic hematopoietic stem cell transplantation (AHSCT) [7, 8] appear to show a better outcome. Altogether, these findings suggest that occult CNS involvement might be already present at diagnosis in a significant fraction of BPDCN patients, and could contribute to explain the relatively high number of CNS recurrences [9].

Here, we prospectively evaluated for the first time the presence of CNS involvement by next generation flow cytometry (NGF) in 13 consecutive BPDCN patients studied at diagnosis (*n* = 10) or at disease recurrence (*n* = 3). The impact of CNS involvement and CNS-directed therapy in patient outcome was further validated in a retrospective series of 23 additional BPDCN patients.

## RESULTS AND DISCUSSION

At diagnosis, most prospectively analyzed patients showed skin lesions (n=10/13; 77%), peripheral blood (7/13; 54%) and/or bone marrow (9/13; 69%) involvement with normal neurological physical examination (n=13/13; 100%) and a good performance status (ECOG≤0/1: n=12/13; 92%) (Table 1). Despite this, cerebrospinal fluid (CSF) samples positive for tumor plasmacytoid dendritic cells were found in 6/10 (60%) cases studied at diagnosis –median of 11 tumor cells/μl (range: 0.6–47), representing 82% of the total CSF cellularity (range: 68%-95%)- in association with ≥20% bone marrow (BM) infiltration by tumor cells (6/6 vs. 1/4 cases, respectively; *p*=.03). Except for the high rate of occult CNS involvement at diagnosis, prospectively analyzed here for the first time, the above described pattern of extranodal disease involvement has been previously reported as typical for BPDCN patients [2, 4, 5].

**Table 1:** BPDCN patients included in the prospective cohort (n=13): Disease features at diagnosis and follow-up including patient outcome

Patient code	Gender	Age	ECOG	Skin lesions	BM % of blast cells at diagnosis	PB % of blast cells at diagnosis	CSF involvement at diagnosis	N. of blast cells/μl detected by NGF in CSF	CC result in CSF^#^	Systemic chemo-therapy	IT therapy at diagnosis	Response to chemo-therapy	Relapse	Site of relapse	Time (months) to relapse/progression	Positive CSF*	Time from diagnosis to IT therapy (days)	Type of IT therapy	N. of IT doses for CSF clearance	CNS relapse/progression	AHSCT	Disease status	Current status	Cause of death	Overall survival (months)
**NO CSF involvement at diagnosis by NGF (n=4)**																									
#3	M	60	1	Yes	29	4	**No**	0	Negative	LAL-07FRAIL	Yes	CR	No			No	2	TIT	NA	No	Yes	CR	Alive		38
#6	F	57	0	Yes	0	0	**No**	0	NE	LAL-AR/2011	Yes	CR	Yes	Cutaneous	7	No	6	TIT	NA	No	Yes	SD	Alive		27
#9	M	67	0	Yes	0	0	**No**	0	NE	LAL-07FRAIL	Yes	CR	No			No	29	TIT	NA	No	No	CR	Alive		20
#11	M	73	0	Yes	2	0	**No**	0	NE	LAL-07FRAIL	Yes	CR	No			No	14	TIT	NA	No	No	CR	Alive		9
Subtotal	3M/1F	64 (57-73)	0	4/4	1 (0-29)	0 (0-4)	**0/4**	0	0/1		4/4	4/4 CR	1/4			0/4	10 (2-29)		NA	0/4	2/4	3/4 CR	4/4 Alive		24 (9-38)
**CSF involvement at diagnosis by NGF (n=6)**																									
#1	M	11	NR	No	80	83	**Yes**	5,1	NE	LAL-AR/1993	Yes	CR	No			Yes	11	TIT	1	No	No	CR	Alive		48
#4	M	16	0	Yes	94	68	**Yes**	0,6	Negative	LAL-AR/2003	Yes	CR	Yes	Leptom	5	Yes	2	TIT & Lip AraC	1	Yes	Yes	CR	Alive		34
#5	M	67	0	Yes	80	45	**Yes**	2,2	Positive	LAL-AR/2011	Yes	CR	No			Yes	3	TIT	1	No	No	CR	EXITUS	Sepsis	2
#10	M	71	1	Yes	88	0	**Yes**	6,4	Negative	LAL-07FRAIL	Yes	CR	No			Yes	5	TIT	4	No	No	CR	Alive		15
#12	F	79	2	No	68	4	**Yes**	46,6	NE	LAL-07FRAIL	Yes	CR	No			Yes	7	TIT & Lip AraC	1	No	No	CR	Alive		7
#13	M	66	0	Yes	74	28	**Yes**	6,1	NE	Hyper-CVAD	Yes	CR	No			Yes	6	TIT	1	No	No	CR	Alive		6
Subtotal	5M/1F	67 (11-79)	0	4/6	80 (68-94)	37 (0-83)	**6/6**	5.6 (0.6-46.6)	1/3		6/6	6/6 CR	1/6			6/6	6 (2-11)		1 (1-44)	1/6	1/6	6/6 CR	5/6 Alive		11 (2-48)
**CSF involvement at relapse/progression by NGF (not evaluated at diagnosis; n=3)**																									
#2	M	58	0	Yes	0	0	**NE**	46,3	NE	No treatment> CHOP at PD	No	PD			14	Yes	426	HD MTX/AraC & Lip AraC	NC	Yes	No	PD	EXITUS	PD	19
#7	M	78	1	No	94	33	**NE**	958,1	NE	CYC & PRED	No	PD			5	Yes	173	NA	NA	Yes	No	PD	EXITUS	PD	5
#8	M	75	0	Yes	0	0	**NE**	4,5	Positive	FLUGAZA	No	SD	Yes	BM, nodal and leptom	7	Yes	226	Lip AraC	1	Yes	No	SD	EXITUS	PD	7
Subtotal	3M/0F	75	0	2/3	0	0	NE	46.3	1/1		0/3	0/3 CR	1/1		7	3/3	226			3/3	0/3	0/3 CR	0/3 Alive		7
**Total n=13**	**11M/2F**	**67 (11-79)**	**0**	**10/13**	**68 (0-94)**	**4 (0-83)**	**6/10**	**6.1 (0.6-958.1)**	**2/5**		**10/13**	**10/13**	**3/11**		**7 (5-14)**	**9/13**	**7 (2-426)**		**1**	**4/13**	**3/13**	**9/13 CR**	**9/13 Alive**		**15 (2-48)**
**Patients that received IT prophylaxis/therapy at diagnosis: Prospective vs validation cohort comparison**																									
Prospective (n=10)	8M/2F	67		8/10	71	4	6/10	5,6	1/4	10 ALL-type	10/10	10/10 CR	2/10	1/2 CNS	6	6/10	6			1/10	3/10	9/10 CR	9/10 Alive		18
Validation (n=5)	3M/2F	31		2/5	73	0	2/2	NE	2/2	4/5 ALL-type	5/5	5/5 CR	2/5	2/2 NR	9	2/5	≤15			0/4	2/5	3/5 CR	3/5 Alive		22
*P* value	NS	0.03		NS	NS	NS	NS			NS	NS	NS	NS		NS	NS	0.01			NS	NS	NS	NS		NS

ECOG: Eastern cooperative oncology group performance status; BM: Bone marrow; PB: Peripheral blood; CSF: Cerebrospinal fluid; NGF: Next generation flow cytometry; CC: Conventional cytology; IT: Intrathecal; CNS: Central nervous system; AHSCT: Allogeneic hematopoietic stem cell transplantation; M: Male; F: Female; NE: Not evaluated; PD: Progressive disease; CYC & PRED: Cyclophosphamide and prednisone; PD: Progressive disease; CR: Complete remission; SD: Stable disease; Leptom: Leptomeningeal; TIT: Triple intrathecal therapy (methotrexate cytarabine and hydrocortisone); Lip: Liposomal; AraC: cytarabine; HD MTX: High dose methotrexate; NA: Not applicable; NC: No clearance of CSF observed; ALL: Acute lymphoblastic leukemia; NR: Not reported; NS: Not statistically significant. #Due to the confirmed superiority of NGF versus CC in detecting occult leptomeningeal disease[12], and the limited volume of available CSF sample, CC evaluation was not systematically performed in parallel *At any time (diagnosis or follow-up).

Patients were grouped according to their CSF status at diagnosis. Results in the summary groups are expressed as number of cases from all cases within the group (for categorical variables) or as median (for continuous variables) and minimum and maximum values.

All the patients had a normal neurological physical examination at diagnosis. 10 were screened for parenchymal involvement with a negative result in all these cases. All but case #7 received IT treatment at some point of the disease but none CNS radiotherapy.

“LAL-XXX” (NCT01358201, NCT00853008 and [21]) and Hyper-CVAD are ALL-type protocols, CHOP is a NHL-type protocol and FLUGAZA (NCT02319135) is an AML-type protocol. CYC & PRED is a palliative regimen.

Regarding the 3 patients referred at relapse (5, 7 and 14 months after diagnosis), none had been screened for CNS disease (nor received CNS therapy) at diagnosis; all 3 had their CSF referred because of the onset of neurological symptoms, consisting of seizure in two patients and the presence of symptoms and signs of intracranial hypertension together with involvement of cranial nerves in the remaining case. They all showed CNS involvement by NGF with >50% tumor cells (958, 5 and 46 tumor cells/μl, respectively). These results confirm previous observations about the relatively high rate of overt CNS involvement in BPDCN, particularly at relapse [1-3]; in addition, these results also support the notion that many BPDCN patients may already have occult CNS disease at diagnosis, in the absence of neurological symptoms.

Despite BPDCN patients have been found to show already at diagnosis, a rather high rate (≈10%) [1-6] of CNS involvement by conventional CSF cytology (e.g. similar to ALL patients), they are neither routinely screened for CNS involvement, nor receive CNS-directed therapy. This is even more striking because CNS relapses have been recurrently reported among BPDCN patients at relapse at higher frequencies than at diagnosis (≈30%) [1-3]. Among other reasons, this might be due to the rarity of the disease –and hence the limited experience with these patients-, as well as the inclusion of BPDCN as a myeloid malignancy in the WHO classification [10], where CNS involvement at diagnosis is uncommon (<5% of patients) [11] and therefore, systematic CSF screening is not performed at diagnosis, in the absence of neurological symptoms. Moreover, NGF has not been routinely applied for the diagnostic screening of CNS involvement in BPDCN patients, despite its higher sensitivity over conventional cytology for detecting tumor cells on stabilized CSF samples [12, 13], and the fact that NGF can detect CNS disease before clinical symptoms emerge [14].

**Figure 1:**
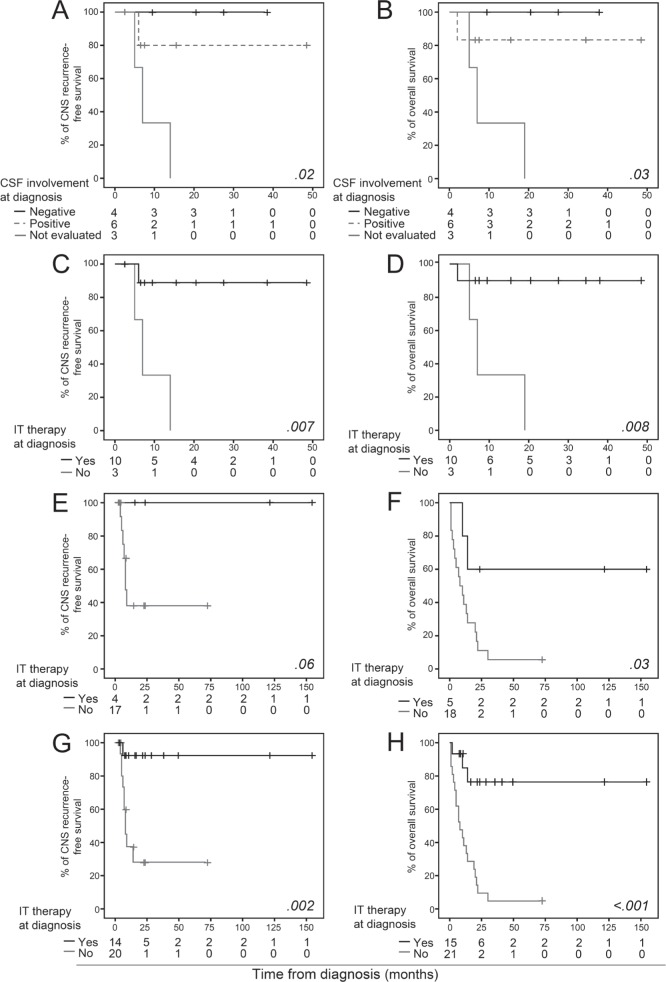
Prognostic impact of (occult) CSF involvement and administration of IT therapy at diagnosis in BPDCN patients CNS-RFS (panels A, C, E and G) and OS (panels B, D, F and H) curves are separately shown for the prospective cohort (panels A, B, C and D), the validation cohort (panels E and F) and the combined (prospective and validation) cohort (panels G and H).

Of note, follow-up CSF samples obtained after triple intrathecal therapy (TIT) showed absence of tumor cells in 6/6 CSF^+^ cases studied at diagnosis, either after one −5/6 cases (83%)- or 4 doses of therapy (6/6 cases; Table 1). At the moment of closing this study, 5/6 cases remained CNS-disease free, in continuous CR; the remaining patient (case#4 in Table 1) had an isolated CNS relapse 6 months after diagnosis. Upon relapse, the patient received intrathecal (IT) chemotherapy, a single dose being sufficient to re-induce CR with NGF-negative CSF; subsequently, he underwent an AHSCT and remains disease-free for >2 years. At the moment of closing this study, only 1/10 patients studied at diagnosis had died in CR (case #5 in Table 1), the other 9 patients remaining alive in CR after a median follow-up of 20 months (range: 6–48 months). In contrast, those three patients studied at disease recurrence suffered disease progression and death, despite IT therapy was administered in 2/3 cases (median overall survival (OS): 7 months) (Table 1). Overall, these findings suggest that BPDCN patients with occult CNS involvement at diagnosis might specifically benefit from CNS-directed therapy, particularly if administered (early) at diagnosis; in line with this, early administration of TIT translated into a significantly better outcome and prolonged survival versus cases who did not receive CNS prophylaxis/therapy at diagnosis and presented with CNS-recurrent disease (Figure 1C and 1D).

In order to further validate our results, a series of 23 BPDCN patients previously reported in the literature [1] -and recruited in an earlier time period-, was retrospectively analyzed; of note, no major differences were observed between the two cohorts (prospectively *vs*. retrospectively analyzed patients) in terms of disease features and outcome (Table 1). Five of these 23 patients received IT prophylaxis at diagnosis and 4/23 received CNS-directed therapy at relapse/progression (Table 2). In this independent validation cohort, patients who did receive IT prophylaxis at diagnosis also had prolonged CNS recurrence-free survival (CNS-RFS) (*p*=.06) and OS (*p*=.03) (Figure 1E and 1F). Univariate analysis of prognostic factors performed in the whole patient cohort (n=36) showed a favorable impact on CNS-RFS and/or OS for children (p>.05; *p*=.03), patients receiving ALL-type therapy (*p*=.01; *p*=.002), AHSCT (*p*>.05; *p*=.003) and IT prophylaxis/treatment at diagnosis (*p*=.002; *p*<.001) (Figure 1G and 1H), the later variable emerging as the only independent (favorable) prognostic factor for CNS-RFS (p=.02, hazard ratio [HR]=11.2, 95% confidence interval [CI]: 1.4 – 88.8) and OS (*p*=.001, HR=7.6, 95% CI: 2.2 – 25.9).

**Table 2:** BPDCN patients included in the validation cohort (n=23): Disease features at diagnosis and follow-up including patient outcome

Patient code	Gender	Age	Skin lesions	BM % of blast cells at diagnosis	PB % of blast cells at diagnosis	CSF involvement at diagnosis	Systemic chemotherapy-type regimen	Response to chemotherapy	Relapse	Site of relapse	Time (months) to relapse/progression	Positive CSF*	Time from diagnosis to IT therapy (days)	CNS treatment	CNS relapse/progression	AHSCT	Current status	Cause of death	Overall survival (months)
**NO CNS IT prophylaxis administered at diagnosis (n=18)**																				
#4R	M	63	No	58	8	**NE**	AML-type	CR	Yes	CNS	6	Yes	183	TIT	Yes	No	EXITUS	PD	10
#6R	M	76	Yes	0	0	**NE**	NHL-type	CR	Yes	Skin	5	No			No	No	EXITUS	PD	13
#8R	M	70	Yes	70	80	**NE**	ALL-type	CR	Yes	CNS, BM	8	Yes	244	TIT & radiotherapy	Yes	No	EXITUS	PD	14
#9R	M	10	Yes	83	15	**NE**	AML-type	CR	Yes	NR	10	No			NR	No	EXITUS	PD	11
#11R	F	34	No	99	0	**NE**	AML-type	CR	No			No			No	Yes	EXITUS	AHSCT complications	7
#19R	M	81	Yes	98	0	**NE**	NHL-type	CR	Yes	CNS, skin and lymph nodes	7	Yes			Yes	No	EXITUS	PD	8
#23R	M	81	Yes	75	0	**NE**	ALL-type	ED				No			No	No	EXITUS	Sepsis	2
#29R	M	78	No	65	35	**NE**	AML-type	ED				No			No	No	EXITUS	TreatTox	1
#31R	M	64	Yes	82	66	**NE**	NHL-type	CR	Yes	CNS, BM	9	Yes	397	TIT & Liposomal AraC	Yes	No	EXITUS	PD	20
#33R	M	72	Yes	81	0	**NE**	NHL-type	NR			4	Yes			Yes	No	EXITUS	PD	4
#38R	M	71	Yes	82	47	**NE**	NHL-type	ED			3	No			No	No	EXITUS	PD	3
#40R	M	79	Yes	25	0	**NE**	NHL-type	CR	Yes	NR	22	No			No	No	EXITUS	PD	22
#42R	M	70	Yes	88	2	**NE**	ALL-type	ED				No			No	No	EXITUS	Sepsis	1
#58R	F	54	Yes	60	40	**NE**	AML-type	ED				No			No	No	EXITUS	TreatTox	1
#59R	M	46	Yes	90	10	**NE**	NHL-type	CR	Yes	CNS	5	Yes			Yes	No	EXITUS	PD	5
#61R	M	52	No	58	12	**NE**	AML-type	CR	No			No			No	Yes	Alive		71
#63R	M	42	No	85	39	**NE**	NHL-type	CR	Yes	CNS, BM	8	Yes	230		Yes	No	EXITUS	PD	30
#64R	F	45	No	75	57	**NE**	AML-type	CR	No			No			No	No	EXITUS	Sepsis	21
Subtotal	15M/3F	67(10-81)	12/18	78(0-99)	11(0-80)	**18 NE**	3/18ALL-type	12/18 CR	9/12	6/9 CNS	7(3-22)	7/18	237(183-397)		7/17	2/18	1/18 Alive		9(1-71)
**CNS IT prophylaxis administered at diagnosis(n=5)**																				
#12R	F	31	No	45	0	**NE**	ALL-type	CR	No			No	≤15	TIT	No	Yes	Alive		153
#13R	M	12	Yes	73	0	**NE**	ALL-type	CR	No			No	≤15	TIT	No	Yes	Alive		22
#28R	M	53	Yes	76	0	**NE**	ALL-type	CR	Yes	NR	10	No	15	MTX & AraC	No	No	EXITUS	PD	14
#35R	M	8	No	85	0	**Yes**	ALL-type	CR	No			Yes	≤15	MTX & Dex	No	No	Alive		120
#44R	F	47	No	73	69	**Yes**	AML-type	CR	Yes	NR	8	Yes	15	AraC	NR	No	EXITUS	PD	10
Subtotal	3M/2F	31(8-53)	2/5	73(45-85)	0(0-69)	**2/2**	4/5 ALL-type	5/5 CR	2/5		9(8-10)	2/5	≤15		0/4	2/5	3/5 Alive		22(10-153)
*P* value^#^	NS	0.02	NS	NS	NS		0.02	NS	NS		NS	NS	0.007		NS	NS	0.02		0.03

BM: Bone marrow; PB: Peripheral blood; CSF: Cerebrospinal fluid; IT: Intrathecal; CNS: Central nervous Sytem; AHSCT: Allogeneic hematopoietic stem cell transplantation; M: Male; F: Female; NE: Not evaluated; ALL: Acute Lymphoblastic Leukemia; NHL: Non-Hodgkin Lymphoma; AML: Acute Myeloblastic Leukemia; CR: Complete remission; ED: Early death; SD: Stable disease; NR: Not reported; TIT: Triple intrathecal therapy (Methotrexate cytarabine and hydrocortisone); AraC: Cytarabine; MTX: Metothrexate; Dex: Dexamethasone; PD: Progressive disease; TreatTox: Treatment toxicity; NS: Not statistically significant.

Results in the summary groups are expressed as number of cases from all cases within the group or as median. *At any time (diagnosis or follow-up) #CNS IT prophylaxis administration at diagnosis versus not.

The high rate of CNS involvement found in our study strongly suggests that the CNS could be a persistent blast-cell sanctuary in BPDCN patients with leukemic presentation, due to the limited power of cytostatic drugs to cross the blood-brain barrier into the CSF and brain parenchyma [15]. This reservoir of leukemic cells may also contribute to BM/systemic disease recurrence. Longer survival rates observed in AHSCT BPDCN patients [7, 8], irrespective of IT medication administration, might be explained by the benefit of the antileukemic effect of donor allogeneic cells [16], able to circulate systemically and cross the blood-brain barrier. Unfortunately, AHSCT is not exempt from relapses (≈1/3 of patients) [7, 8], it cannot overcome the poor disease status beyond the first CR [7], and toxicity-related mortality is high [17]. Additionally, many patients are elderly or unfit subjects to undergo such an intensive therapeutic approach.

Recently, SL-401, a novel targeted therapy directed to the interleukin-3 receptor, has shown positive results on BPDCN patients with skin confined disease, whereas long-term benefit has not yet been observed in patients with the leukemic variant/phase of the disease [18]. No comparison can be established in terms of CNS relapses between both approaches (SL-401 *vs*. IT prophylaxis) since patients showing CNS involvement are excluded from the study (NCT02113982) and sites of relapse/progression after SL-401 administration have not been detailed [18, 19]. Nonetheless, evaluation of IT injection of this targeted therapy will be of great interest, particularly in CNS relapses.

To the best of our knowledge, this is the first study reporting a strikingly high frequency of occult CNS involvement at diagnosis in BPDCN patients, and the potential benefit of CNS-directed therapy to reverse the poor outcome of BPDCN patients,. Indirectly, our results also suggest that occult CNS disease might contribute to explain the higher frequency of recurrences (e.g. CNS) observed in these patients, despite their high CR rates. Further prospective studies on larger BPDCN patient cohorts studied at diagnosis are necessary to confirm our findings.

## MATERIALS AND METHODS

### Ethics statement

The study was approved by the Ethics Committee of the Cancer Research Center, and performed following the Declaration of Helsinki. Each participant gave his/her informed consent prior entering the study.

### Patients and samples

Forty-one CSF samples from 13 consecutive BPDCN patients (11 males and 2 females; median age: 67 years, range: 11-79 years) were evaluated for the presence of CNS involvement by NGF. Cases were evaluated at diagnosis (*n* = 10) or at relapse/progression (*n* = 3) and subsequently, after IT therapy. Diagnosis of BPDCN was based on previously reported recommendations [10], including expression of markers claimed to be mandatory to allocate blast cells to the plasmacytoid dendritic cell lineage [20], by either flow cytometry or immunohistochemistry of peripheral blood specimens, BM samples or cutaneous lesions. The 10 patients studied at diagnosis received high-risk ALL-type treatment, based on the Spanish PETHEMA protocols (NCT01358201, NCT00853008 and [21])(*n* = 9) or HyperCVAD therapy (*n* = 1), including one dose of TIT as CNS prophylaxis at each treatment phase (Table 1). For CSF-positive cases, additional IT treatment was given -TIT every 72h (*n* = 4) or liposomal cytarabine (50mg) every two weeks (*n* = 2)- until two consecutive CSF-negative samples were obtained. In turn, patients with recurrent disease (*n* = 3) received AML-type or NHL-type (CHOP) therapy (Table 1).

In order to validate the impact of CNS involvement and CNS-directed therapy on patient outcome, an independent validation cohort of 23 BPDCN treated with ALL- (*n* = 7), AML- (*n* = 8) or NHL-type (*n* = 8) therapeutic regimens was retrospectively analyzed [1]. The intrathecal treatment administered to these patients is detailed in Table 2.

Multiparameter NGF immunophenotypic studies were performed on Transfix™-stabilized CSF samples (Cytomark, Buckingham, UK) following the EuroFlow panels and protocols [12, 22]. Data analysis was performed with the Infinicyt software (Cytognos, Salamanca, Spain). The lower cut-off for CNS involvement was defined as a cluster ≥10 events with the appropriate phenotype, based on a 10-parameter tube (≥0.001 cells/μl).

### Statistical analyses

The Mann-Whitney U (for continuous variables) and the χ^2^tests (for categorical variables) were used to determine the statistical significance of differences observed between groups (PASW 19 statistical software, IBM SPSS Statistics, IBM, Armonk, NY, USA). OS and CNS-RFS curves were plotted according to the method of Kaplan-Meier and compared using the (one-sided) log-rank test. Those variables showing prognostic value in the univariate analysis were also evaluated by multivariate analysis using a Cox stepwise regression model. Statistically significance was set at p values < .05.
